# The relationship between target-class and the physicochemical properties of antibacterial drugs

**DOI:** 10.1016/j.bmc.2015.04.063

**Published:** 2015-08-15

**Authors:** Grace Mugumbate, John P. Overington

**Affiliations:** European Molecular Biology Laboratory—European Bioinformatics Institute (EMBL-EBI), Wellcome Trust Genome Campus, Hinxton, CB10 1SD, United Kingdom

**Keywords:** Antibacterials, Physicochemical properties, Drug targets, Ribosome

## Abstract

The discovery of novel mechanism of action (MOA) antibacterials has been associated with the concept that antibacterial drugs occupy a differentiated region of physicochemical space compared to human-targeted drugs. With, in broad terms, antibacterials having higher molecular weight, lower log *P* and higher polar surface area (PSA). By analysing the physicochemical properties of about 1700 approved drugs listed in the ChEMBL database, we show, that antibacterials for whose targets are riboproteins (i.e., composed of a complex of RNA and protein) fall outside the conventional human ‘drug-like’ chemical space; whereas antibacterials that modulate bacterial protein targets, generally comply with the ‘rule-of-five’ guidelines for classical oral human drugs. Our analysis suggests a strong target-class association for antibacterials—either protein-targeted or riboprotein-targeted. There is much discussion in the literature on the failure of screening approaches to deliver novel antibacterial lead series, and linkage of this poor success rate for antibacterials with the chemical space properties of screening collections. Our analysis suggests that consideration of target-class may be an underappreciated factor in antibacterial lead discovery, and that in fact bacterial protein-targets may well have similar binding site characteristics to human protein targets, and questions the assumption that larger, more polar compounds are a key part of successful future antibacterial discovery.

## Graphical abstract

**Figure:**
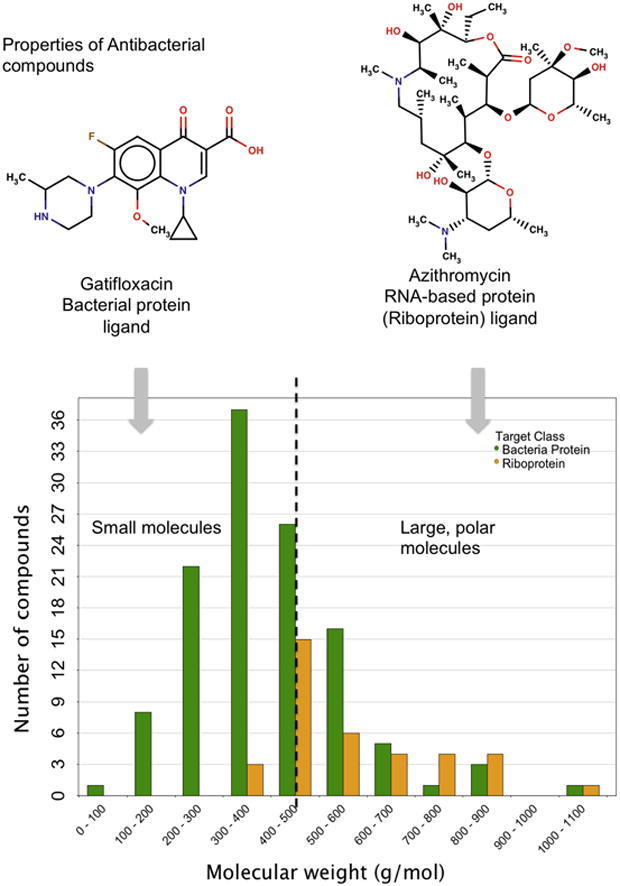

## 1 Introduction

The discovery of antibacterial agents, such as the aminoglycosides, streptogramins, tetracyclines, β-lactams, and quinolones, from the 1930s onwards^1^ brought significant relief to the health burden caused by pathogenic bacteria worldwide. Nevertheless, the success of antibacterial chemotherapy is now being significantly challenged by the emergence of drug-resistant bacterial strains, poor hit rates from genomics/target-led screens^2^ and high drug failure rates in late clinical development.^3^ Treatment of bacterial infectious diseases is also often complicated by comorbidity, for example, co-infection with human immunodeficiency virus (HIV), extended duration of chemotherapy and diagnostic delays.^4^ Drug-resistant strains have a direct impact on the clinical treatment of diseases such as leprosy, *Staphylococcus*, *Strepto*- and *Enterococcus*, *Clostridia*, and *Pseudomonas*, and arguably most significantly on tuberculosis (TB).

Tuberculosis and leprosy are mycobacterial diseases affecting substantial parts Africa, the Americas, South-East Asia, the Eastern Mediterranean and the Western Pacific Regions and together cause close to a million deaths every year.^5,6^ To curb the development of resistance to rifampicin, multidrug therapy (MDT) is often used, where the drug is used in combination with other antibacterials. MDT has been successful as evidenced by the decrease in the number of reported leprosy or TB cases but it increases the drug burden on the patient, and could lead to non-compliance and the eventual emergence of resistance. In the industrialised Northern hemisphere drug resistance, for example with hospital-acquired methicillin-resistant *Staphylococcus aureus* (MRSA), is also perceived as a major societal threat. The aforementioned challenges call for continual discovery and development of new mechanism differentiated antibacterials. These drugs would potentially be less prone to resistance (or at least have distinguished resistance profiles), have new modes of action and/or targets and with the capability to reduce drug load.

Advances in technologies in genomics and sequencing, high-throughput screening (HTS), automated chemical synthesis and structural biology, have so far had limited direct impact on the discovery of new classes of drugs; it has taken more than four decades for the novel linezolid class of antibacterial drugs (2000), to be approved^7,1^, and more recently bedaquiline (2012) for treatment of drug-resistant tuberculosis.^8^ This productivity challenge has drawn researchers in industry and academia, and more recently funding agencies and public bodies, to propose and develop new approaches to antibacterial discovery. Examples include identification of whole cell-based methods to identify antibacterial leads with the ability to penetrate the bacterial cell wall, in conjunction with currently used target-based approaches. Furthermore, the consideration of the physicochemical properties of the chemical compounds during the early stages of drug discovery process^9,1,10^ has helped to avoid failure in Phases II/III of clinical development. Experimental and computational approaches have therefore been used to analyse the historical physicochemical properties of drugs and to develop models used in determining drug-like compounds, and in some cases, propose biased or focussed properties for antibacterials.^11^

Study of the properties of approved drug structures in the 1990’s led to the concept of a restricted range of physicochemical parameters that appeared optimal for orally administered drugs—many researchers have used the ‘rule of five’^12^ as a guide for ‘drug-like’ compounds during screening and structure modification of leads to optimise their potency, metabolism and exposure. The ‘rule of five’ states that good absorption is more likely when an orally administered compound has less than five hydrogen-bond donors, molecular weight <500 Da, *c* log *P* <5, and less than 10 hydrogen-bond acceptors.^12,13^ Later it was also observed that polar surface area (PSA) and *a* log *P* could predict with 95% confidence that a test compound would have high or low (∼90% or ∼30%, respectively) intestinal absorption in humans.^14^ For high absorption the upper limits were, PSA 131.6 Å^2^, and *a* log *P* of 5.88. Veber et al.^15^ suggested that compounds with 10 or less rotatable bonds and PSA less than or equal to 140 Å^2^ (or 12 or fewer hydrogen-bond donors or acceptors) would have good oral availability in rats. A number of authors subsequently reported that approved antibacterial agents often violated Lipinski’s ‘rule of five’, with antibacterials occupying a different region of physicochemical space^7,9,16^, some of which are dosed parenterally or topically and are more polar and have high aqueous solubility.^8^

Recently the physicochemical space of antibacterials has been classified based on bacteria classes by O’Shea et al.^7^ For 147 active antibacterial compounds analysed, it was shown that Gram-positive and Gram-negative antibacterials have different physicochemical properties; for example, the mean molecular weights were 813 and 414, respectively. These findings were attributed to the different cell-wall architecture of the two bacterial classes. Both classes contain an inner membrane and a peptidoglycan layer. In addition, Gram-negative bacteria consist of an outer less permeable polar membrane and promiscuous efflux pumps.^7,10^ The first attempt to classify antibacterials agents based on target-classes was made by Brown et al.^10^ They showed that physical properties of HTS antibacterial hits were associated with their respective targets. For example, in *Pseudomonas aeruginosa*, compounds binding to the hydrophobic pocket of UDP-(3-*O*-(*R*-hydroxymyristoyl))-*N*-acetylglucosamine deacetylase (LpxC) were generally hydrophobic. This analysis focused mostly on Gram-negative HTS hits and conclusions were drawn based on one bacterial organism. On the other hand, Leeson et al.^17^ reported that for most human drugs, lipophilicity was comparable indicating the importance of this parameter irrespective of the drug’s target, but a different pattern was observed for several anti-infectives including antibacterials.

The scope of our current work is based on the hypothesis that the different physicochemical properties of antibacterial agents may also be related to the nature and type of their molecular targets responsible for drug efficacy. Here we focus on differences in the surface properties of the binding pockets for bacterial-proteins (where the molecular target is either simple protein, protein families or protein–protein complex) and riboproteins (where the molecular target is an RNA or RNA/protein species). We analysed the physicochemical properties of about 2000 compounds consisting of world-wide approved antibacterial agents targeting either proteins or riboproteins and non-antibacterials targeting specifically human proteins, using analysis routines implemented in Pipeline Pilot (Biovia).^18^ The goal was to determine the association of physicochemical properties of antibacterials and their target-class. Bioactive compounds that target human proteins generally fall within the established parameter range for Lipinski ‘drug-like’ compounds and were therefore used as comparator benchmarks.

## 2 Methods

### 2.1 Source of target-ligand pairs

All compound and target data used in this work was obtained from the ChEMBL database^19,20^ an Open Access database of bioactive small molecules and activity data. ChEMBL version 19, holds ∼1.4 million distinct bioactive small molecules, ∼10 thousand annotated targets, and more than 12 million activities. The data has been manually extracted from about 57,000 publications and curated by experienced chemistry and biology curators.

### 2.2 Drugs and their targets dataset

A dataset containing more than 1700 drugs including bioactivity information was retrieved from ChEMBL version 19 using a Pipeline Pilot protocol (Biovia).^18^ An SQL query was written to pull out information for distinct approved drugs and their annotated targets. The criteria used included extraction of simple drug molecules that were approved drugs, and had a specified therapeutic application (therapeutic flag 1).

### 2.3 Physicochemical properties and analysis

**Figure 1:**
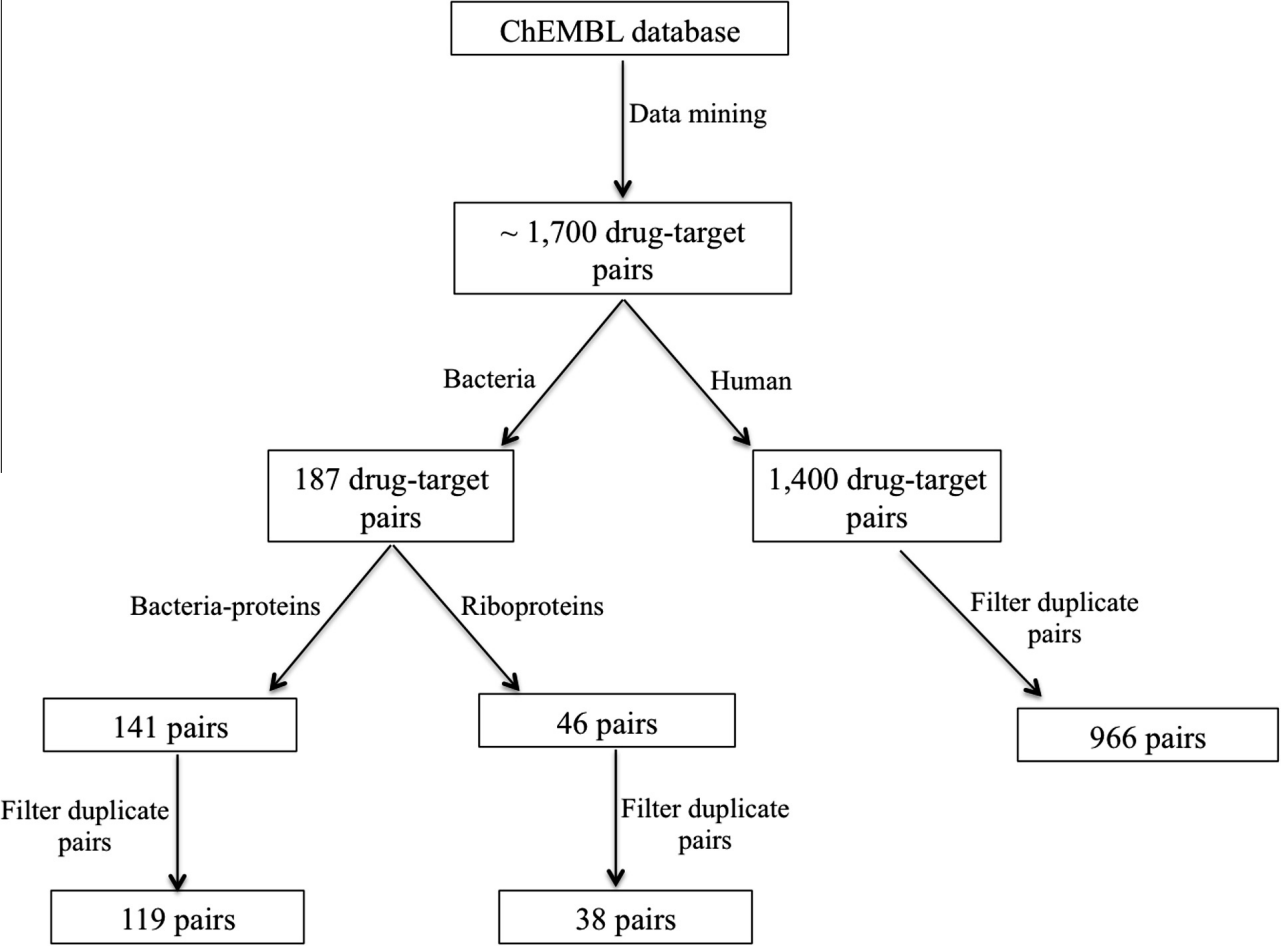
A workflow used to mine target-ligand data from the ChEMBL version 19. The dataset was split into three subsets containing ligands targeting human protein, bacterial-protein and bacterial riboproteins targets.

The dataset was split into two, the first subset consisted of drugs that act through human targets and the second one had those that act through bacterial targets. The bacterial target subset was further divided into two specific sub-subsets; namely (i) bacterial-proteins (where the molecular target is a simple molecule, protein family or a protein–protein complex) and riboproteins (where the molecular target is an RNA or RNA/protein species). We identified no possibility to split the human set into RNA and protein subsets, since no evidence of specific RNA-class targets were identifiable in ChEMBL. For each target, duplicate molecules were filtered from these three sets, so that in each only unique drug molecules were analysed (Fig. 1). Various simple physicochemical properties including molecular weight (Mwt), *a* log *P*, log *D*, polar surface area (PSA), H-bond donors and H-bond acceptors were calculated using the standard components in Pipeline Pilot. Analysis of the physicochemical properties of ‘human proteins’, ‘bacterial proteins’ and ‘bacterial riboproteins’ ligand sets was performed using Vortex from Dotmatics Ltd (http://www.dotmatics.com/products/vortex/).

## 3 Results and discussion

A total of 1713 target-ligand pairs of drugs that target proteins from 42 organisms, including human, bacteria, plasmodia, viruses, was mined from the ChEMBL database (www.ebi.ac.uk/chembl).^20^ Human target-ligand pairs constituted ∼82% (1419 pairs) of the set, bacteria pairs made up ∼11% (187 pairs). Comparison of the physicochemical properties of antibacterials indicates a relationship between the compound properties and the class of proteins they target.

**Figure 2:**
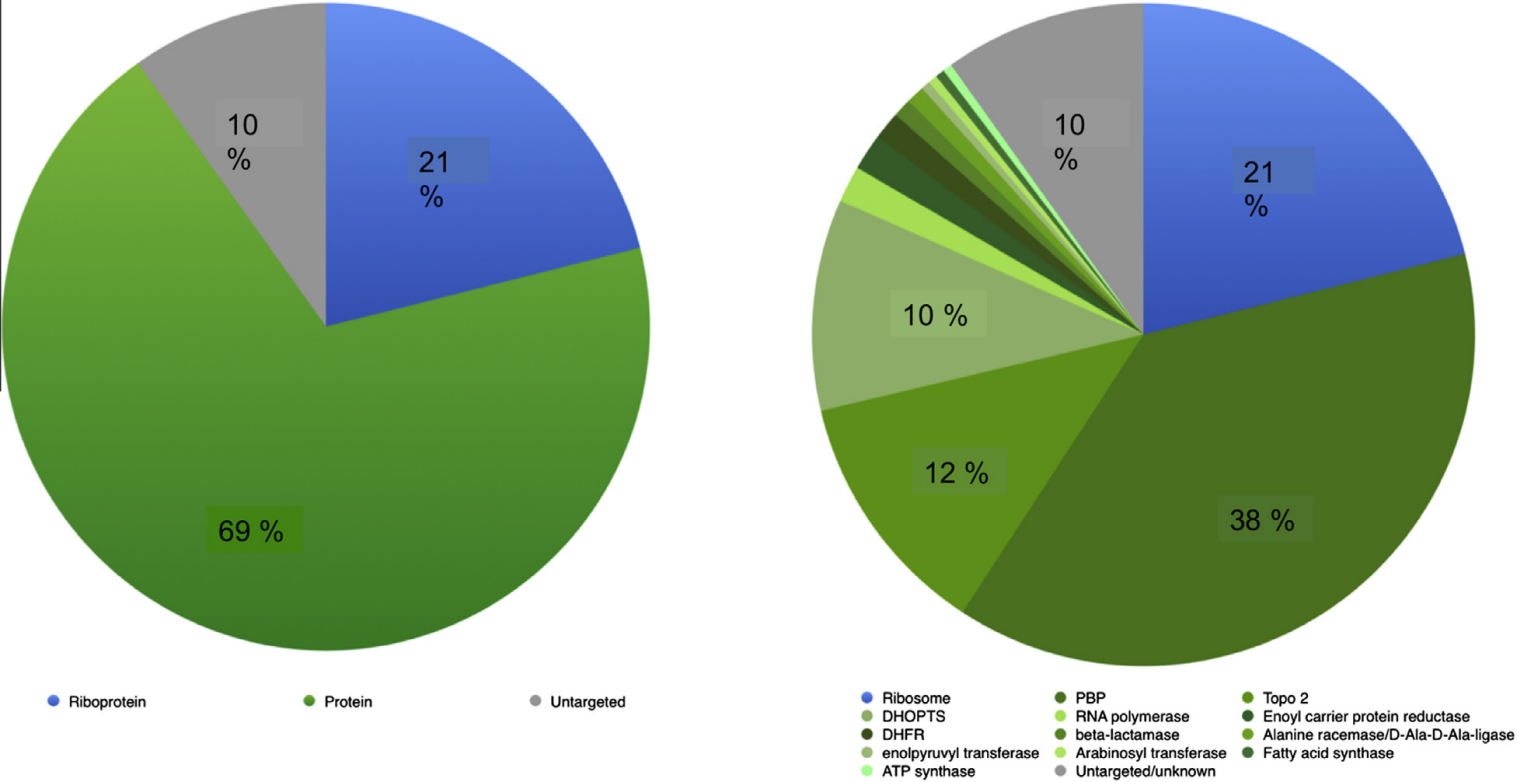
Results for the ATC classification of antibacterials indicating that most drugs target bacterial-proteins.

The target-ligand dataset was initially divided into four classes made up of (i) single proteins, for example bacterial enoyl-[acyl-carrier-protein] reductase, a target for one of the first-line anti-tuberculosis drugs, isoniazid; (ii) protein families, homologous proteins with high sequence similarity, for example, bacterial penicillin binding proteins (PBPs) modulated by drugs like doripenem; (iii) protein complexes, for example, topoisomerase IV targeted by fluoroquinolones, such as gatifloxacin or (iv) RNA–protein complexes (riboproteins), for example, the 70S ribosome that contains a large number of structurally distinct binding sites for approved drugs, such as erythromycin. This classification indicated that about three quarters of the approved antibacterials act upon single proteins, protein families and other protein complexes and only ∼25% target ribosomes (Table 1). A similar analysis of a set of approved antibacterials was retrieved using the WHO Anatomical Therapeutic Chemical (ATC)^21^ classification codes J01 and J04 (available from http://www.whocc.no/atc_ddd_index/). The letter J indicates that a drug is an anti-infective for systematic use, J01 covers antibacterials for systematic use, and J04 antimycobacterials. The analysis of WHO ATC drugs (a broader set than the FDA-approved core of ChEMBL drugs) revealed comparable proportions as 69% antibacterial drugs target bacterial-proteins whilst ∼21% target riboproteins as shown in Figure 2.

There is a general and significant increase in the average of each property from single proteins ligands to compounds targeting riboproteins (Table 1). The single protein class is mainly targeted by sulfonamides that are small and on average have properties within the typical range of oral drugs. The same is true for penicillins and fluoroquinolones, nonetheless the riboproteins-targeted ligands occupy a different physicochemical space characterised by higher molecular weight (average Mwt = 566), high polarity (PSA of 193), and with number of H-bond acceptor and donors larger than the ‘drug-like’ threshold. In contrast, the human protein set of ligands is small (average Mwt <400), slightly lipophilic, and less polar and as discussed above, none of these are known to target nucleic-acid complexes.

### 3.1 Comparison of physicochemical properties of bacterial-protein and riboprotein ligands

#### 3.1.1 Molecular weight

**Figure 3:**
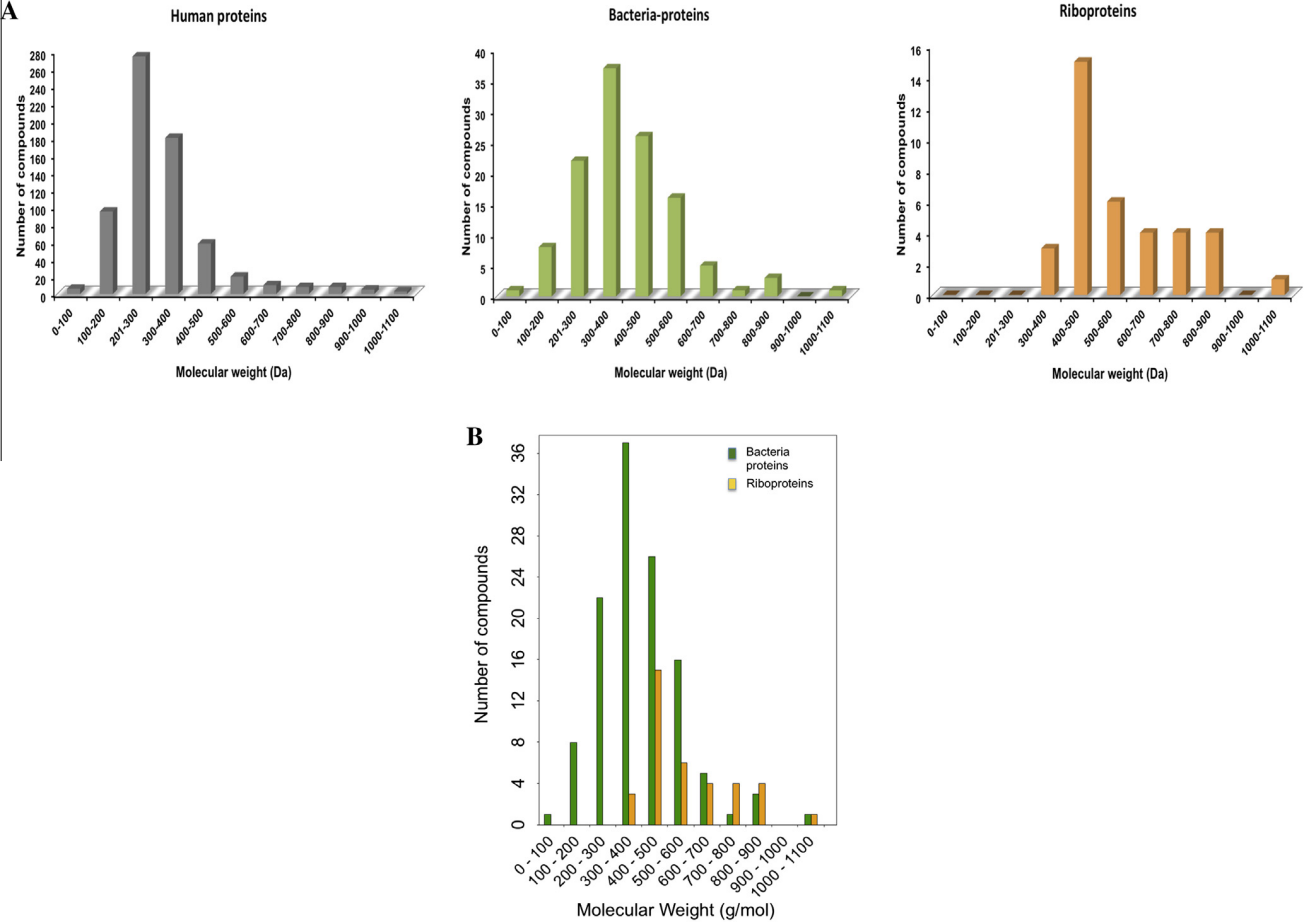
(A) Distribution of molecular weights (Mwt) for riboprotein, human and bacterial-protein targeting drugs. (B) The distribution of molecular weight of bacterial-protein and bacterial-riboprotein compounds.

A comparison of the simple but fundamental property, molecular weight, of antibacterials, indicates that about 80% (30/38) of the drugs whose modes of action involve riboproteins (ribosomes) (orange) have Mwt >500 Da and some of the ligands e.g. quinupristin, have Mwt over 1000 Da. Whereas ∼87% (105/120) of unique bacterial protein ligands (green) had Mwt <500 Da, a region also occupied by about 93% (900/966) of ligands for human proteins (grey) (Fig. 3). Most bacterial protein targeting molecules occupy the same physicochemical space (Mwt <500 Da)^10^ as orally administered drugs from other therapeutic areas even though the medians of molecular weights of bacterial and human protein modulators differ by ∼40 units, (380 and 340, respectively). The slight increase in molecular weights for bacterial protein ligands could be attributed to the presence of the rifamycins, such as rifampicin (Mwt: 822.94 Da), that target DNA-directed RNA polymerase^22^ and contain a privileged macrocyclic ring. Further analysis of Figure 3 indicates that there is no normal distribution the Mwts. From Wilcoxon test, at significant level *p* = 0.05, it is possible to suggest that there no difference between human and bacterial sets (*p* = 0.57). A *p*-value greater than 0.05 confirms the null hypothesis, which states that there is no significant difference the medians of the two sets.

On the contrary, the set of riboprotein ligands displayed a median ∼500 Da and the molecular weight increases gradually from ∼120 to 1022 Da, as shown in Figure 3. The smallest putative ligand, pyrazinamide (Mwt = 123 Da)^23,24^, whereas the largest compound, quinupristin (Mwt = 1040 Da) is a streptogramin that blocks the translation of mRNA into protein.^25^ The calculated *p*-values from the Wilcoxon test for Mwt distribution of human/riboprotein sets (*p* = 4.68e–08), and bacterial/riboprotein sets (*p* = 6.42e–04) indicate that riboprotein targeted compounds occupy a different molecular weight space. Based on the molecular weights the riboprotein targeting compounds constitute two major classes of compounds: small molecular size group made up primarily of tetracyclines (400 > Mwt < 500), and a large molecular size group consisting mostly macrolides and aminoglycosides.

#### 3.1.2 Lipophilicity and polarity

**Figure 4:**
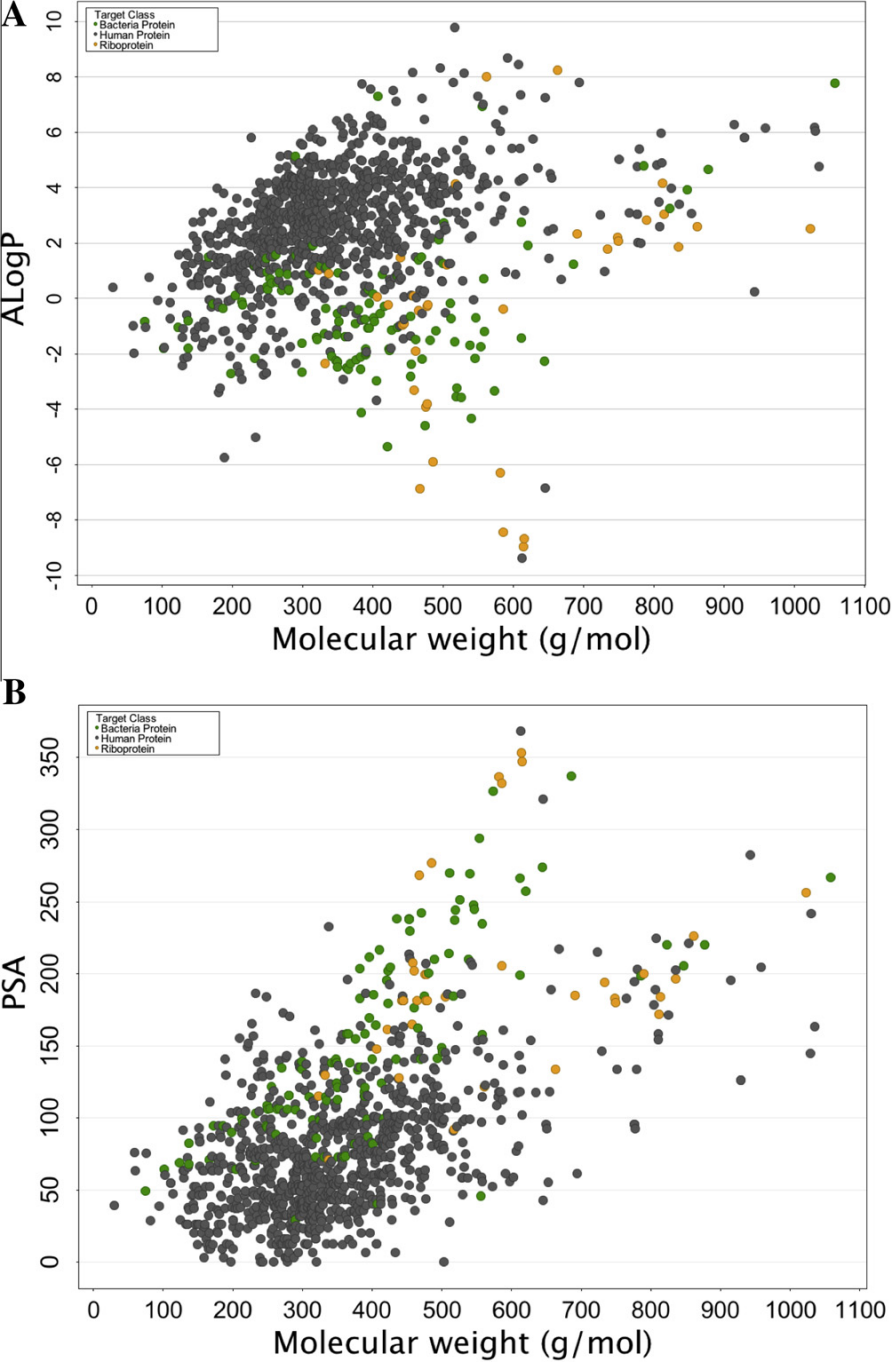
Distribution of (A) *a* Log *P*, and (B) PSA against molecular weight for human, bacterial-proteins and bacterial-riboproteins.

Antibacterials are collectively classified as large and polar compounds with relatively low lipophilicity.^7,9^ The 2D distributions in Figure 4A of calculated *a* log *P* values and Mwt indicated that bacterial-protein and bacterial-riboprotein modulators are less lipophilic than human protein ligands. From the statistical analysis, it can be suggested that there is no significant value between the bacterial and riboprotein datasets (*p* = 0.63) but significant difference is displayed by the human/bacteria and human/riboprotein sets (*p* <0.05). However, riboprotein ligands displayed much lower lipophilicity with approximately 19% of the total compounds showing *a* log *P* >2 (Fig. 4) and low calculated log *D* values (Supporting information, Fig. S1). Compounds with the lowest lipophilicity were three aminoglycosides, neomycin (Mwt = 614.16 Da, *a* log *P* = −8.96), amikacin (Mwt = 585.6 Da, *a* log *P* = −8.43), and paromomycin (Mwt = 615.63 Da, *a* log *P* = −8.67) that bind to pockets in the 30S subunit of the bacterial ribosome. Aminoglycosides form an important class of antibacterials used to treat a variety of infections but display poor attributes for oral absorption, hence, they are mainly administered intravenously, for example, amikacin. Paromomycin, formerly used to treat intestinal infections has been repositioned and is currently used as an injectable in chemotherapy of protozoal visceral leishmaniasis in India.^26^

An opposite but similar trend was revealed by the calculate PSA values where most antibacterial compounds were generally highly polar. Approximately 92% of the human protein targeting drugs exhibited PSA values below 140 Å^2^, a recommended limit for good oral absorption.^15^ Bacterial-protein modulators displayed moderate polarity (∼45% compounds have PSA <130 Å^2^), whilst 80% of riboprotein ligands showed high polarity (PSA >130 Å^2^) indicating, once again, a difference between the two target-classes. Statistically all three datasets have significant difference in PSA distribution, since the calculated Wilcoxon *p*-values were less than 0.05. Further analysis of the results revealed that there was significant correlation between *a* log *P* and log *D* (*r*^2^ = 0.72), and Mwt and PSA (*r*^2^ = 0.91) for bacterial-protein ligands, whereas low or poor correlation is displayed by riboproteins target properties except for log *D* and PSA (*r*^2^ = 0.98) (Supporting information, Fig. S1). This shows that increase in molecular weight of the ligands does not directly influence lipophilicity across both target-classes as previously reported.^17^ Contrary to the properties of riboproteins targeted drugs, for small antibacterials, polarity of a compound increases with increase in the molecular weight.

#### 3.1.3 Riboprotein binding drugs

Following the landmark determination of complete, high-resolution structures^27,28^ of the bacterial ribosome, for which a Nobel prize was awarded, there is now detailed understanding of distinct drug binding sites, resistance mechanisms, and the possibility to design new agents using structural approaches. The ribosomes consist of two subunits—30S inhibited by tetracyclines and the aminoglycosides, and the 50S subunit, acted upon by, for example macrolides, Ribosome binding pockets are generally large with width greater than 10 Å and provide nucleotide rich environments that are highly polar due to presence of the phosphate groups in the RNA backbone, and the higher fractional content of polar N and O atoms in the nucleotide bases compared to amino acid side chains, H-bond acceptor and donor atoms, in addition to hydrophobic moieties.^29,30^ Consequently, tetracyclines (Support information, Table S2) are made up of fused ring systems with highly polar groups on one side that form a dense network of hydrogen bonds with a magnesium ion and nucleotide residues found in the primary site of 30S ribosome.^29^ The hydrophobic edge of the drug molecule is conducive for hydrophobic interactions or stacking interactions with the aromatic rings of the binding site residues. Similarly, macrolides, for example, erythromycin, consist of the lactone ring where C5 atom is bonded to a sugar residues at C5 atom. Ribosome-inhibitor interactions are enhanced through hydrophobic interactions with the lactone ring and hydrophilic interactions involving the sugar substructures.^30^ This illustrates that for strong and effective binding to their targets, the designed small molecules should match the architecture of the binding sites of their respective targets. Normally, hydrophobic molecules bind to hydrophobic binding sites through dispersion forces and pi–pi interactions for example. On the other hand, large and polar molecules would form hydrogen bonds, ionic interactions and salt-bridges with the respective binding site residues. Accordingly, riboprotein targeting compounds have exceptional physicochemical properties that favour binding to the main ribosome binding sites. These observations highlight the fact that the observed difference in molecular properties for the two classes of antibacterial drugs examined here (protein or riboproteins targeted) are due to the distinct differences in binding site properties driven by differences in composition and polarity of the underlying constituent biopolymers.

## 4 Conclusion

Most antibacterials that act via bacterial protein targets have physicochemical properties within the classical range for oral human protein targeted drugs, whereas a majority of the bacterial riboprotein-targeted drugs fall beyond the specified upper limit of the Rule of Five—500 Da (Mwt), and are more polar. We propose therefore that it is useful to consider antibacterial compounds as falling into three classes; (i) bacterial protein-targeted ligands (small and moderately lipophilic), (ii) riboprotein-targeted ligands (large molecular sizes and high polarity and these generally violate the ‘Rule of Five’), and (iii) non-gene product-targeted ligands (e.g., compounds such as gramicidin). We believe that this treatment would add value to the future discovery and optimisation of antibacterials, and that combining target-class and binding site information with appropriate physicochemical parameters models would facilitate drug discovery, especially during screening file design and acquisition, target prediction, hit identification and lead optimisation stages. Finally, the extrapolation of antibacterial drug property profiles derived independent of target class and applying them to bacterial protein targets, as typically found in genetic knockout and bioinformatics studies may well decrease the productivity of antibacterial drug discovery. Conversely collection of compounds that are likely to bind to riboproteins surfaces may well be an excellent strategy for discovery of novel ribosome modulators, an already comprehensively validated antibacterial drug target.

## Figures and Tables

**Supplementary Figure 6:**
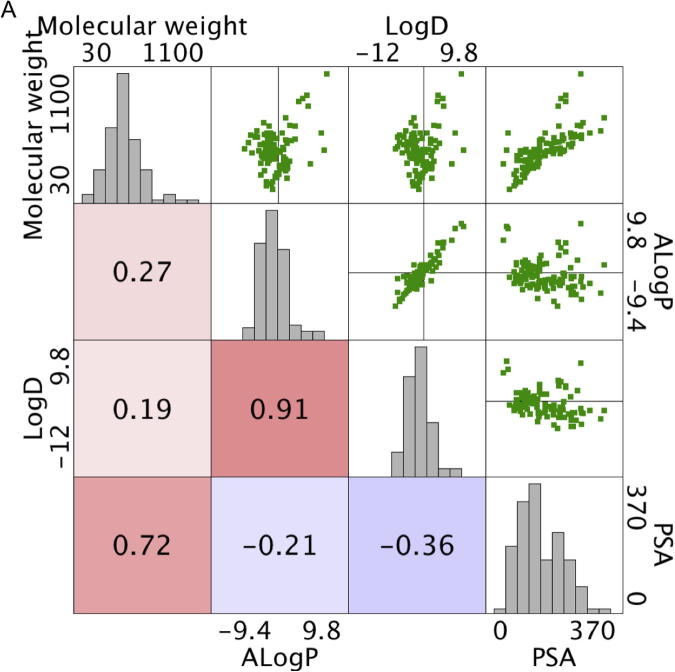
Figure S1A.

**Supplementary Figure 7:**
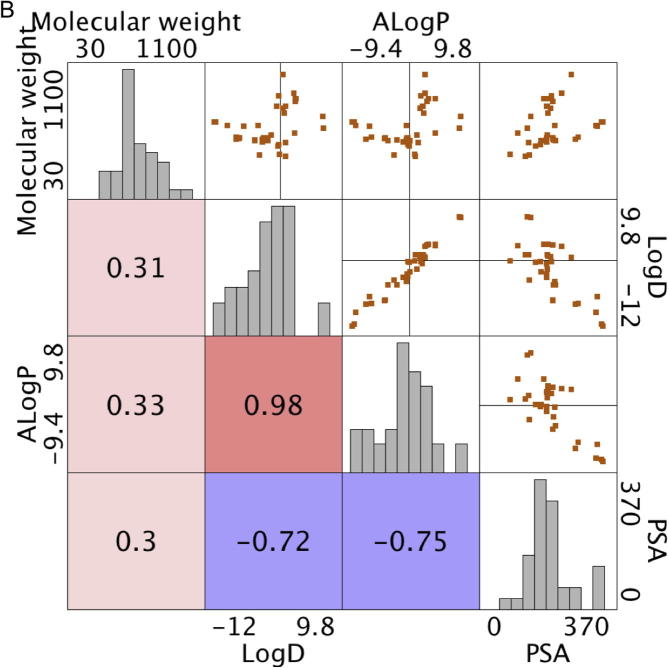
Figure S1B.

**Table 1:** Classes of bacterial targets and the mean property of their ligands

Target-class	No. of compds	Examples of compd class	Molecular weight (Da)	*A* log *P*	PSA	# H-bond donors	# H-bond acceptors
Single proteins	33		261	0.50	101	2	5
Protein families	62		445	−1.44	190	2	9
Protein complex	24		312	0.44	79	2	6
Riboprotein	38		566	−0.31	193	5	11

## Supplementary data

Supplementary data This document contains supplementary materials.
