# Supplementary material for: The relationship between target-class and the physicochemical properties of antibacterial drugs
Source: Bioorg Med Chem. 2015 Aug 15;23(16):5218–24. doi: 10.1016/j.bmc.2015.04.063 (PMC4537081; doi:10.1016/j.bmc.2015.04.063)

**Supporting Information**

The relationship between target-class and physicochemical properties of antibacterials drugs.

Grace Mugumbate and John P. Overington^*^

European Molecular Biology Laboratory – European Bioinformatics Institute (EMBL-EBI), Wellcome Trust Genome Campus, Hinxton, CB10 1SD, United Kingdom

^*^Corresponding author: [jpo@ebi.ac.uk](mailto:jpo@ebi.ac.uk), Tel: 00 44 (0) 1223 494467

**Contents Page**

Table 1: List of antibacterials 2

Table 2: Examples of compounds targeting riboproteins 18

**Figure S1:** Matrix showing correlation between molecular weight, PSA, aLogP and LogD 20

**Table 1: List of Antibacterial Compounds**

| Number | Compound Name | Target name | Target Class | Mwt  g/mol | AlogP | PSA | H-bond Acceptor | H-bond donor | LogD |
| --- | --- | --- | --- | --- | --- | --- | --- | --- | --- |
| 1 | RETAPAMULIN | 70S ribosome | RNA | 517.76 | 4.15 | 92.14 | 6 | 1 | -6.542 |
| 2 | LINEZOLID | 70S ribosome | RNA | 337.35 | 0.89 | 71.11 | 5 | 1 | -1.927 |
| 3 | TOBRAMYCIN | 70S ribosome | RNA | 467.51 | -6.86 | 268.17 | 14 | 10 | 0.233 |
| 4 | LINCOMYCIN HYDROCHLORIDE | 70S ribosome | RNA | 406.54 | 0.07 | 147.79 | 8 | 5 | -2.276 |
| 5 | TROLEANDOMYCIN | 70S ribosome | RNA | 813.97 | 3.04 | 184.18 | 16 | 0 | -6.717 |
| 6 | ETHAMBUTOL HYDROCHLORIDE | Arabinosyltransferase A | Protein | 204.31 | 0.11 | 64.52 | 4 | 4 | -1.418 |
| 7 | BEDAQUILINE FUMARATE | ATP synthase | Protein | 555.5 | 6.93 | 45.59 | 4 | 1 | -10.636 |
| 8 | CHLORAMPHENICOL SODIUM SUCCINATE | Bacterial 70S ribosome | RNA | 422.19 | -0.23 | 161.57 | 8 | 2 | -5.062 |
| 9 | QUINUPRISTIN | Bacterial 70S ribosome | RNA | 1022.22 | 2.52 | 256.5 | 14 | 4 | -8.34 |
| 10 | OXYTETRACYCLINE CALCIUM | Bacterial 70S ribosome | RNA | 458.42 | -3.3 | 207.51 | 10 | 5 | -2.802 |
| 11 | ERYTHROMYCIN ESTOLATE | Bacterial 70S ribosome | RNA | 789.99 | 2.84 | 199.98 | 15 | 4 | -5.559 |
| 12 | ERYTHROMYCIN ETHYLSUCCINATE | Bacterial 70S ribosome | RNA | 862.05 | 2.59 | 226.27 | 17 | 4 | -5.795 |
| 13 | TETRACYCLINE HYDROCHLORIDE | Bacterial 70S ribosome | RNA | 444.43 | -0.9 | 181.61 | 9 | 6 | -2.76 |
| 14 | CLINDAMYCIN PALMITATE HYDROCHLORIDE | Bacterial 70S ribosome | RNA | 663.39 | 8.25 | 133.63 | 8 | 3 | -10.568 |
| 15 | ERYTHROMYCIN LACTOBIONATE | Bacterial 70S ribosome | RNA | 733.93 | 1.79 | 193.91 | 14 | 5 | -4.759 |
| 16 | NEOMYCIN SULFATE | Bacterial 70S ribosome | RNA | 614.64 | -8.96 | 353.11 | 19 | 13 | 0.439 |
| 17 | DALFOPRISTIN | Bacterial 70S ribosome | RNA | 690.85 | 2.33 | 184.8 | 10 | 2 | -6.499 |
| 18 | METHACYCLINE HYDROCHLORIDE | Bacterial 70S ribosome | RNA | 442.42 | -0.95 | 181.62 | 9 | 6 | -3.388 |
| 19 | AZITHROMYCIN | Bacterial 70S ribosome | RNA | 748.98 | 2.08 | 180.08 | 14 | 5 | -4.516 |
| 20 | CHLORAMPHENICOL PALMITATE | Bacterial 70S ribosome | RNA | 561.54 | 8 | 121.44 | 6 | 2 | -11.282 |
| 21 | OXYTETRACYCLINE HYDROCHLORIDE | Bacterial 70S ribosome | RNA | 460.43 | -1.9 | 201.85 | 10 | 7 | -2.784 |
| 22 | KANAMYCIN SULFATE | Bacterial 70S ribosome | RNA | 485.48 | -5.91 | 276.82 | 15 | 11 | 1.187 |
| 13 | TIGECYCLINE | Bacterial 70S ribosome | RNA | 585.65 | -0.38 | 205.76 | 11 | 7 | -4.09 |
| 24 | CLARITHROMYCIN | Bacterial 70S ribosome | RNA | 747.95 | 2.2 | 182.91 | 14 | 4 | -5.383 |
| 25 | AMIKACIN SULFATE | Bacterial 70S ribosome | RNA | 585.6 | -8.43 | 331.93 | 17 | 13 | -1.30E-02 |
| 26 | CHLORAMPHENICOL | Bacterial 70S ribosome | RNA | 323.13 | 1.02 | 115.38 | 5 | 3 | -4.117 |
| 27 | NETILMICIN SULFATE | Bacterial 70S ribosome | RNA | 475.58 | -3.9 | 199.72 | 12 | 8 | -1.306 |
| 28 | STREPTOMYCIN SULFATE | Bacterial 70S ribosome | RNA | 581.57 | -6.29 | 336.43 | 19 | 12 | -1.322 |
| 29 | PAROMOMYCIN SULFATE | Bacterial 70S ribosome | RNA | 615.63 | -8.67 | 347.32 | 19 | 13 | 0.614 |
| 30 | DIRITHROMYCIN | Bacterial 70S ribosome | RNA | 835.07 | 1.87 | 196.33 | 16 | 5 | -5.217 |
| 31 | TELITHROMYCIN | Bacterial 70S ribosome | RNA | 812 | 4.17 | 171.84 | 13 | 1 | -7.953 |
| 32 | MINOCYCLINE HYDROCHLORIDE | Bacterial 70S ribosome | RNA | 457.48 | 0.1 | 164.63 | 9 | 5 | -2.77 |
| 33 | MECLOCYCLINE SULFOSALICYLATE | Bacterial 70S ribosome | RNA | 476.86 | -0.28 | 181.62 | 9 | 6 | -4.235 |
| 34 | CLINDAMYCIN PHOSPHATE | Bacterial 70S ribosome | RNA | 504.96 | 1.21 | 183.9 | 10 | 5 | -2.751 |
| 35 | CLINDAMYCIN HCL | Bacterial 70S ribosome | RNA | 439.01 | 1.48 | 127.56 | 7 | 4 | -3.766 |
| 36 | GENTAMICIN SULFATE | Bacterial 70S ribosome | RNA | 477.6 | -3.81 | 199.72 | 12 | 8 | -1.427 |
| 37 | SPECTINOMYCIN HYDROCHLORIDE | Bacterial 70S ribosome | RNA | 332.35 | -2.33 | 129.51 | 9 | 5 | -0.284 |
| 38 | DEMECLOCYCLINE HYDROCHLORIDE | Bacterial 70S ribosome | RNA | 464.85 | -0.43 | 181.62 | 9 | 6 | -3.376 |
| 39 | CHLORTETRACYCLINE HYDROCHLORIDE | Bacterial 70S ribosome | RNA | 478.88 | -0.23 | 181.61 | 9 | 6 | -3.543 |
| 40 | CLAVULANATE POTASSIUM | Bacterial beta-lactamase TEM | Protein | 198.15 | -2.71 | 89.9 | 5 | 1 | -2.6 |
| 41 | SULBACTAM SODIUM | Bacterial beta-lactamase TEM | Protein | 232.23 | -2.15 | 102.96 | 5 | 0 | -1.52 |
| 42 | TAZOBACTAM SODIUM | Bacterial beta-lactamase TEM | Protein | 299.28 | -2.66 | 133.66 | 7 | 0 | -1.19 |
| 43 | TRIMETHOPRIM SULFATE | Bacterial dihydrofolate reductase | Protein | 290.32 | 1.54 | 105.51 | 7 | 2 | 1.54 |
| 44 | SULFAMETER | Bacterial dihydropteroate synthase | Protein | 280.3 | 0.3 | 115.58 | 6 | 2 | -0.13 |
| 45 | SULFADIAZINE, SILVER | Bacterial dihydropteroate synthase | Protein | 249.27 | 0.42 | 108.41 | 5 | 1 | 0.31 |
| 46 | SULFAMERAZINE | Bacterial dihydropteroate synthase | Protein | 264.3 | 0.59 | 106.35 | 5 | 2 | 0.6 |
| 47 | SULFALENE | Bacterial dihydropteroate synthase | Protein | 280.3 | 0.33 | 115.58 | 6 | 2 | 0.41 |
| 48 | SULFANILAMIDE | Bacterial dihydropteroate synthase | Protein | 172.2 | -0.21 | 94.55 | 3 | 2 | -0.21 |
| 49 | SULFATHIAZOLE | Bacterial dihydropteroate synthase | Protein | 255.32 | 0.57 | 121.69 | 4 | 2 | 0.57 |
| 50 | SULFACETAMIDE | Bacterial dihydropteroate synthase | Protein | 214.24 | -0.22 | 97.64 | 4 | 2 | -0.12 |
| 51 | SULFADIAZINE | Bacterial dihydropteroate synthase | Protein | 250.28 | 0.31 | 106.35 | 5 | 2 | 0.31 |
| 52 | SULFACETAMIDE SODIUM | Bacterial dihydropteroate synthase | Protein | 213.23 | -0.12 | 99.71 | 4 | 1 | -0.12 |
| 53 | MAFENIDE ACETATE | Bacterial dihydropteroate synthase | Protein | 186.23 | -0.36 | 94.55 | 3 | 2 | -1.2 |
| 54 | SULFAPYRIDINE | Bacterial dihydropteroate synthase | Protein | 249.29 | 0.96 | 93.46 | 4 | 2 | 0.95 |
| 55 | SULFAMETHIZOLE | Bacterial dihydropteroate synthase | Protein | 270.33 | 0.3 | 134.59 | 5 | 2 | 0.41 |
| 56 | SULFISOXAZOLE DIOLAMINE | Bacterial dihydropteroate synthase | Protein | 267.3 | 0.92 | 106.6 | 4 | 2 | 1.02 |
| 57 | SULFAPHENAZOLE | Bacterial dihydropteroate synthase | Protein | 314.36 | 1.92 | 98.38 | 4 | 2 | 1.93 |
| 58 | SULFAMETHOXAZOLE | Bacterial dihydropteroate synthase | Protein | 253.28 | 0.71 | 106.6 | 4 | 2 | 0.8 |
| 59 | SULFAMETHAZINE | Bacterial dihydropteroate synthase | Protein | 278.33 | 0.88 | 106.35 | 5 | 2 | 0.88 |
| 60 | SULFISOXAZOLE ACETYL | Bacterial dihydropteroate synthase | Protein | 309.34 | 0.91 | 114.88 | 5 | 1 | 0.49 |
| 61 | NALIDIXIC ACID | Bacterial DNA gyrase | Protein | 232.24 | 1.18 | 70.5 | 5 | 1 | 0.26 |
| 62 | LOMEFLOXACIN HYDROCHLORIDE | Bacterial DNA gyrase | Protein | 351.35 | -0.83 | 72.88 | 6 | 2 | -0.2 |
| 63 | CIPROFLOXACIN HYDROCHLORIDE | Bacterial DNA gyrase | Protein | 331.34 | -1.27 | 72.88 | 6 | 2 | -1.36 |
| 64 | MOXIFLOXACIN HYDROCHLORIDE | Bacterial DNA gyrase | Protein | 401.43 | -0.7 | 82.11 | 7 | 2 | -0.68 |
| 65 | CINOXACIN | Bacterial DNA gyrase | Protein | 262.22 | 1.57 | 88.43 | 7 | 1 | 1.52 |
| 66 | OFLOXACIN | Bacterial DNA gyrase | Protein | 361.37 | -1.37 | 73.32 | 7 | 1 | 1.48 |
| 67 | SPARFLOXACIN | Bacterial DNA gyrase | Protein | 392.4 | -1.06 | 98.9 | 7 | 3 | -1.04 |
| 68 | RIFABUTIN | Bacterial DNA-directed RNA polymerase | RNA | 847 | 3.93 | 205.54 | 14 | 5 | 3.5 |
| 69 | RIFAMPICIN | Bacterial DNA-directed RNA polymerase | RNA | 822.94 | 3.26 | 220.15 | 15 | 6 | 2.54 |
| 70 | RIFAXIMIN | Bacterial DNA-directed RNA polymerase | RNA | 785.88 | 4.8 | 198.37 | 12 | 5 | 3.73 |
| 71 | RIFAPENTINE | Bacterial DNA-directed RNA polymerase | Protein | 877.03 | 4.66 | 220.15 | 15 | 6 | 2.74 |
| 72 | TRICLOSAN | Bacterial enoyl-[acyl-carrier-protein] reductase | Protein | 289.54 | 5.14 | 29.46 | 2 | 1 | 5.13 |
| 73 | HEXACHLOROPHENE | Bacterial enoyl-[acyl-carrier-protein] reductase | Protein | 406.9 | 7.31 | 40.46 | 2 | 2 | 7.3 |
| 74 | CEFTIBUTEN | Bacterial penicillin-binding protein | Protein | 410.42 | -0.54 | 216.46 | 9 | 4 | -3 |
| 75 | AMPICILLIN SODIUM | Bacterial penicillin-binding protein | Protein | 348.4 | -1.29 | 140.86 | 6 | 2 | -2.28 |
| 76 | MEROPENEM | Bacterial penicillin-binding protein | Protein | 383.46 | -4.13 | 135.48 | 7 | 3 | -3.85 |
| 77 | PENICILLIN G POTASSIUM | Bacterial penicillin-binding protein | Protein | 333.38 | -0.35 | 114.84 | 5 | 1 | -0.35 |
| 78 | CEFIXIME | Bacterial penicillin-binding protein | Protein | 453.45 | -0.65 | 238.05 | 11 | 4 | -3.6 |
| 79 | CEFPROZIL HYDRATE | Bacterial penicillin-binding protein | Protein | 389.43 | -2.03 | 158.26 | 7 | 4 | -1.22 |
| 80 | CEFACLOR HYDRATE | Bacterial penicillin-binding protein | Protein | 367.81 | -2.33 | 138.02 | 6 | 3 | -2.32 |
| 81 | CEPHAPIRIN SODIUM | Bacterial penicillin-binding protein | Protein | 422.46 | -1.81 | 179.33 | 9 | 1 | -1.26 |
| 82 | CEFMENOXIME HYDROCHLORIDE | Bacterial penicillin-binding protein | Protein | 511.56 | -0.71 | 269.64 | 13 | 3 | -2.19 |
| 83 | MOXALACTAM DISODIUM | Bacterial penicillin-binding protein | Protein | 518.46 | -3.55 | 237.26 | 13 | 2 | -3.54 |
| 84 | CEFDITOREN PIVOXIL | Bacterial penicillin-binding protein | Protein | 620.72 | 1.93 | 257.18 | 12 | 2 | 1.93 |
| 85 | CEFMETAZOLE SODIUM | Bacterial penicillin-binding protein | Protein | 470.53 | -2.18 | 242.05 | 12 | 1 | -2.18 |
| 86 | CEFPIRAMIDE SODIUM | Bacterial penicillin-binding protein | Protein | 611.63 | -1.43 | 266.19 | 13 | 4 | -1.43 |
| 87 | TICARCILLIN DISODIUM | Bacterial penicillin-binding protein | Protein | 382.41 | -2.56 | 183.21 | 7 | 1 | -2.55 |
| 88 | CARBENICILLIN DISODIUM | Bacterial penicillin-binding protein | Protein | 376.38 | -2.22 | 154.97 | 7 | 1 | -2.22 |
| 89 | CEFAMANDOLE NAFATE | Bacterial penicillin-binding protein | Protein | 489.5 | -1.17 | 210.03 | 11 | 1 | -1.17 |
| 90 | IMIPENEM HYDRATE | Bacterial penicillin-binding protein | Protein | 299.35 | -1.62 | 139.02 | 6 | 4 | -2.94 |
| 91 | DORIPENEM | Bacterial penicillin-binding protein | Protein | 420.5 | -5.34 | 195.74 | 8 | 5 | -5.12 |
| 92 | LORACARBEF HYDRATE | Bacterial penicillin-binding protein | Protein | 349.77 | -2.48 | 112.73 | 5 | 3 | -2.46 |
| 93 | PENICILLIN V | Bacterial penicillin-binding protein | Protein | 350.39 | 0.92 | 121.24 | 6 | 2 | -0.55 |
| 94 | PIPERACILLIN SODIUM | Bacterial penicillin-binding protein | Protein | 516.55 | -1.56 | 184.55 | 8 | 2 | -1.56 |
| 95 | PENICILLIN V POTASSIUM | Bacterial penicillin-binding protein | Protein | 349.38 | -0.55 | 124.07 | 6 | 1 | -0.55 |
| 96 | PENICILLIN G BENZATHINE | Bacterial penicillin-binding protein | Protein | 334.39 | 1.12 | 112.01 | 5 | 2 | -0.35 |
| 97 | CEFTIZOXIME SODIUM | Bacterial penicillin-binding protein | Protein | 382.39 | -2.12 | 203.58 | 9 | 2 | -2.12 |
| 98 | CEFORANIDE | Bacterial penicillin-binding protein | Protein | 519.55 | -3.23 | 244.22 | 12 | 4 | -4.69 |
| 99 | CEFAZOLIN SODIUM | Bacterial penicillin-binding protein | Protein | 453.5 | -2.81 | 237.76 | 11 | 1 | -2.81 |
| 100 | CEPHALEXIN HYDRATE | Bacterial penicillin-binding protein | Protein | 347.39 | -2.25 | 138.02 | 6 | 3 | -2.25 |
| 101 | CEFUROXIME SODIUM | Bacterial penicillin-binding protein | Protein | 423.38 | -1.88 | 201.89 | 9 | 2 | -1.87 |
| 102 | AMPICILLIN | Bacterial penicillin-binding protein | Protein | 349.4 | -2.3 | 138.03 | 6 | 3 | -2.28 |
| 103 | CEFDINIR | Bacterial penicillin-binding protein | Protein | 395.41 | -0.17 | 211.75 | 9 | 4 | -1.65 |
| 104 | AMOXICILLIN | Bacterial penicillin-binding protein | Protein | 365.4 | -2.54 | 158.26 | 7 | 4 | -1.73 |
| 105 | BACAMPICILLIN HYDROCHLORIDE | Bacterial penicillin-binding protein | Protein | 465.52 | 1.4 | 162.56 | 9 | 2 | 1.27 |
| 106 | CEFUROXIME AXETIL | Bacterial penicillin-binding protein | Protein | 510.47 | -0.18 | 214.35 | 11 | 2 | -0.18 |
| 107 | CEFADROXIL | Bacterial penicillin-binding protein | Protein | 363.39 | -2.5 | 158.26 | 7 | 4 | -1.69 |
| 108 | MEZLOCILLIN SODIUM | Bacterial penicillin-binding protein | Protein | 538.57 | -1.69 | 210 | 9 | 2 | -1.69 |
| 109 | CARBENICILLIN INDANYL SODIUM | Bacterial penicillin-binding protein | Protein | 493.55 | 2.12 | 141.14 | 7 | 1 | 2.12 |
| 110 | CEFOPERAZONE SODIUM | Bacterial penicillin-binding protein | Protein | 644.66 | -2.27 | 273.69 | 13 | 3 | -2.27 |
| 111 | CEFOTAXIME SODIUM | Bacterial penicillin-binding protein | Protein | 454.46 | -2.38 | 229.88 | 11 | 2 | -2.39 |
| 112 | CEPHRADINE | Bacterial penicillin-binding protein | Protein | 349.4 | -2.28 | 138.02 | 6 | 3 | -2.27 |
| 113 | CEFOTETAN DISODIUM | Bacterial penicillin-binding protein | Protein | 573.6 | -3.33 | 326.78 | 15 | 2 | -3.26 |
| 114 | CEFTAZIDIME SODIUM | Bacterial penicillin-binding protein | Protein | 545.57 | -2.17 | 247.58 | 11 | 2 | -2.14 |
| 115 | CEFTRIAXONE SODIUM | Bacterial penicillin-binding protein | Protein | 553.57 | -1.75 | 294.14 | 15 | 3 | -1.75 |
| 116 | CEFPODOXIME PROXETIL | Bacterial penicillin-binding protein | Protein | 557.6 | 0.71 | 234.5 | 13 | 2 | 0.71 |
| 117 | CEFTAROLINE FOSAMIL ACETATE | Bacterial penicillin-binding protein | Protein | 685.69 | 1.23 | 337.29 | 14 | 5 | -1.73 |
| 118 | CEFEPIME HYDROCHLORIDE | Bacterial penicillin-binding protein | Protein | 481.57 | -1.5 | 200.75 | 9 | 3 | -2.98 |
| 119 | CEPHALOGLYCIN | Bacterial penicillin-binding protein | Protein | 405.42 | -2.97 | 164.33 | 8 | 3 | -2.96 |
| 120 | AZTREONAM | Bacterial penicillin-binding protein | Protein | 435.43 | -1.03 | 238.19 | 11 | 4 | -4.73 |
| 121 | CEFOXITIN SODIUM | Bacterial penicillin-binding protein | Protein | 426.44 | -1.15 | 204.62 | 8 | 2 | -1.15 |
| 122 | CEPHALOTHIN SODIUM | Bacterial penicillin-binding protein | Protein | 395.43 | -1.07 | 169.38 | 7 | 1 | -1.07 |
| 123 | CYCLACILLIN | Bacterial penicillin-binding protein | Protein | 341.43 | -2.09 | 138.03 | 6 | 3 | -2.08 |
| 124 | CEFONICID SODIUM | Bacterial penicillin-binding protein | Protein | 540.55 | -4.33 | 269.54 | 13 | 2 | -4.31 |
| 125 | CEFTAZIDIME | Bacterial penicillin-binding protein | Protein | 546.58 | -0.69 | 244.75 | 11 | 3 | -2.14 |
| 126 | ERTAPENEM SODIUM | Bacterial penicillin-binding protein | Protein | 474.51 | -4.6 | 184.4 | 9 | 4 | -4.38 |
| 127 | CEFOTIAM HYDROCHLORIDE | Bacterial penicillin-binding protein | Protein | 525.63 | -3.56 | 251.3 | 12 | 3 | -3.56 |
| 128 | AMDINOCILLIN | Bacterial penicillin-binding protein 2 | Protein | 325.43 | 1.19 | 98.51 | 6 | 1 | -0.39 |
| 129 | ACETOHYDROXAMIC ACID | Bacterial urease | Protein | 75.07 | -0.82 | 49.33 | 2 | 2 | -0.82 |
| 130 | CYCLOSERINE | D-alanylalanine synthetase | Protein | 102.09 | -1.79 | 64.34 | 3 | 2 | -1.79 |
| 131 | DAPSONE | Dihydropteroate synthase 1 | Protein | 248.3 | 1.44 | 94.55 | 4 | 2 | 1.44 |
| 132 | SULFOXONE SODIUM | Dihydropteroate synthase 1 | Protein | 402.47 | -0.82 | 185.26 | 8 | 2 | -0.82 |
| 133 | NOVOBIOCIN SODIUM | DNA gyrase | Protein | 611.62 | 2.75 | 198.92 | 11 | 4 | 2.75 |
| 134 | FIDAXOMICIN | DNA-directed RNA polymerase | RNA | 1058.04 | 7.77 | 266.65 | 18 | 7 | 6.98 |
| 135 | ISONIAZID | Enoyl-[acyl-carrier-protein] reductase | Protein | 137.14 | -0.81 | 68 | 3 | 2 | -0.71 |
| 136 | ETHIONAMIDE | Enoyl-[acyl-carrier-protein] reductase | Protein | 166.24 | 1.5 | 71 | 2 | 1 | 1.54 |
| 137 | PYRAZINAMIDE | Fatty acid synthase | Protein | 123.11 | -1.04 | 68.87 | 3 | 1 | -1.04 |
| 138 | MUPIROCIN CALCIUM | Isoleucyl-tRNA synthetase | RNA | 499.61 | 1.26 | 148.87 | 9 | 3 | 1.29 |
| 139 | MUPIROCIN | Isoleucyl-tRNA synthetase | RNA | 500.62 | 2.74 | 146.04 | 9 | 4 | 1.29 |
| 140 | DICLOXACILLIN SODIUM | Penicillin-binding protein | Protein | 469.32 | 1.49 | 140.87 | 6 | 1 | 2.96 |
| 141 | HETACILLIN POTASSIUM | Penicillin-binding protein | Protein | 388.46 | -0.74 | 118.08 | 6 | 1 | -0.26 |
| 142 | NAFCILLIN SODIUM | Penicillin-binding protein | Protein | 413.47 | 0.86 | 124.07 | 6 | 1 | 0.86 |
| 143 | HETACILLIN | Penicillin-binding protein | Protein | 389.47 | -1.97 | 115.25 | 6 | 2 | -0.26 |
| 144 | CLOXACILLIN SODIUM | Penicillin-binding protein | Protein | 434.87 | 0.83 | 140.87 | 6 | 1 | 2.3 |
| 145 | AZLOCILLIN SODIUM | Penicillin-binding protein | Protein | 460.48 | -1.47 | 176.28 | 7 | 3 | -1.47 |
| 146 | METHICILLIN SODIUM | Penicillin-binding protein | Protein | 379.41 | -0.42 | 133.29 | 7 | 1 | -0.42 |
| 147 | OXACILLIN SODIUM | Penicillin-binding protein | Protein | 400.43 | 0.16 | 140.87 | 6 | 1 | 1.63 |
| 148 | GEMIFLOXACIN MESYLATE | Topoisomerase IV | Protein | 389.38 | -1.83 | 121.35 | 9 | 2 | -0.24 |
| 149 | GATIFLOXACIN | Topoisomerase IV | Protein | 375.39 | -0.91 | 82.11 | 7 | 2 | -0.89 |
| 150 | ENOXACIN | Topoisomerase IV | Protein | 320.32 | -1.48 | 85.77 | 7 | 2 | -1.02 |
| 151 | LEVOFLOXACIN | Topoisomerase IV | Protein | 361.37 | -1.37 | 73.32 | 7 | 1 | 1.48 |
| 152 | ALATROFLOXACIN MESYLATE | Topoisomerase IV | Protein | 558.51 | -1.18 | 157.95 | 9 | 4 | -0.59 |
| 153 | TROVAFLOXACIN MESYLATE | Topoisomerase IV | Protein | 416.35 | -0.27 | 99.76 | 7 | 2 | -0.04 |
| 154 | NORFLOXACIN | Topoisomerase IV | Protein | 319.33 | -1.41 | 72.88 | 6 | 2 | -0.42 |
| 155 | GREPAFLOXACIN HYDROCHLORIDE | Topoisomerase IV | Protein | 359.39 | -0.41 | 72.88 | 6 | 2 | -0.39 |
| 156 | BESIFLOXACIN HYDROCHLORIDE | Topoisomerase IV | Protein | 393.84 | 0.32 | 86.87 | 6 | 2 | 0.45 |
| 157 | FOSFOMYCIN TROMETHAMINE | UDP-N-acetylglucosamine 1-carboxyvinyltransferase | Protein | 137.05 | -1.79 | 82.7 | 4 | 1 | -1.96 |

Table 2: Examples of compounds targeting riboproteins

| Compound class | Structures | |
| --- | --- | --- |
| Tetracyclines |   Tetracycline | Tigecycline |
|  | Oxytetracycline | Meclocycline |
| Macrolides | Erythromycin | Clarithromycin |

**Figure S1:** Matrix showing correlation between molecular weight, PSA, aL ogP and LogD for A) bacteria-protein ligands and B) Riboprotein ligands. Ranges for the property values are also indicated. Relative counts of compounds at a given value are shown by the histograms and scatter plots indicate the distribution of each property with respect to another.


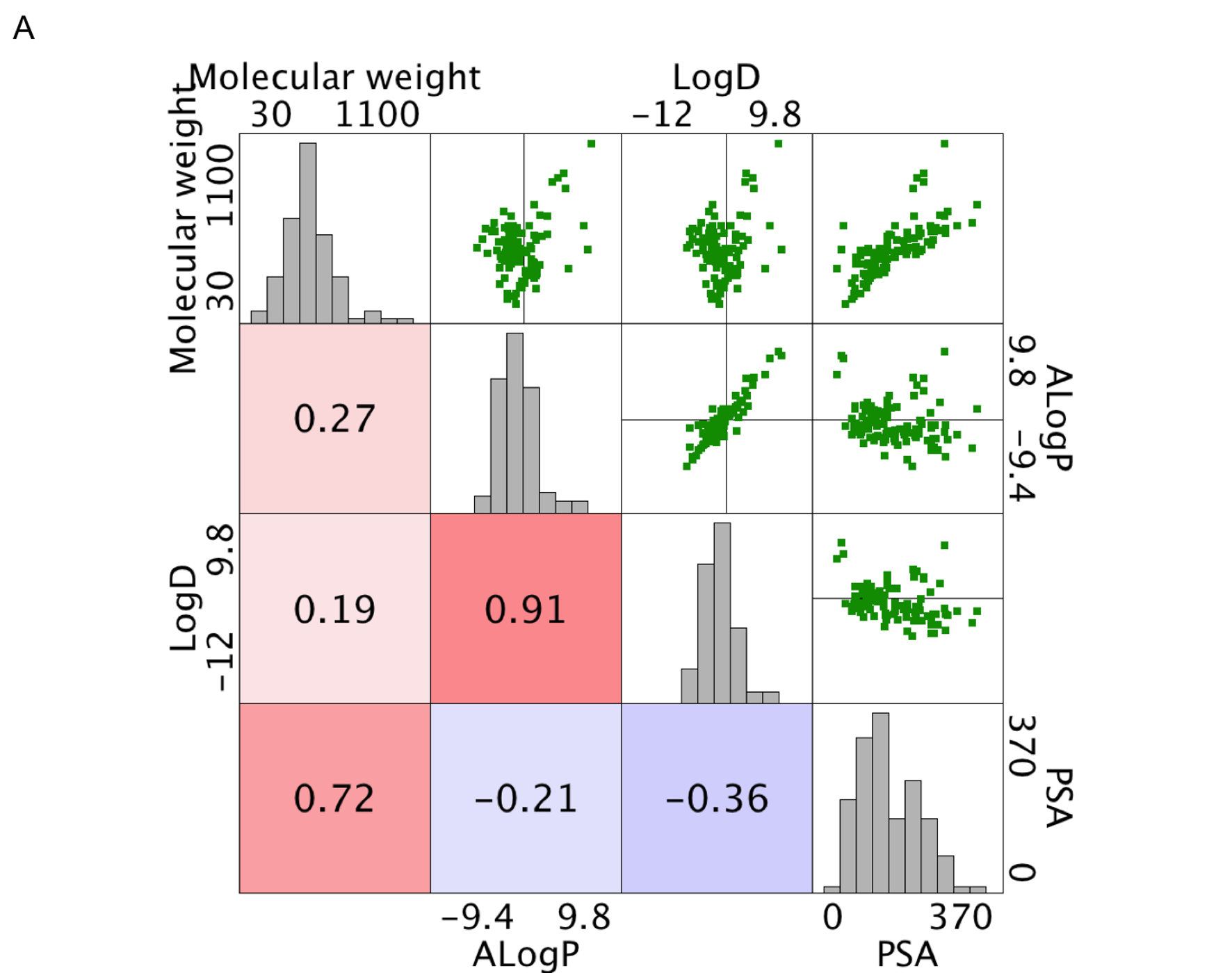

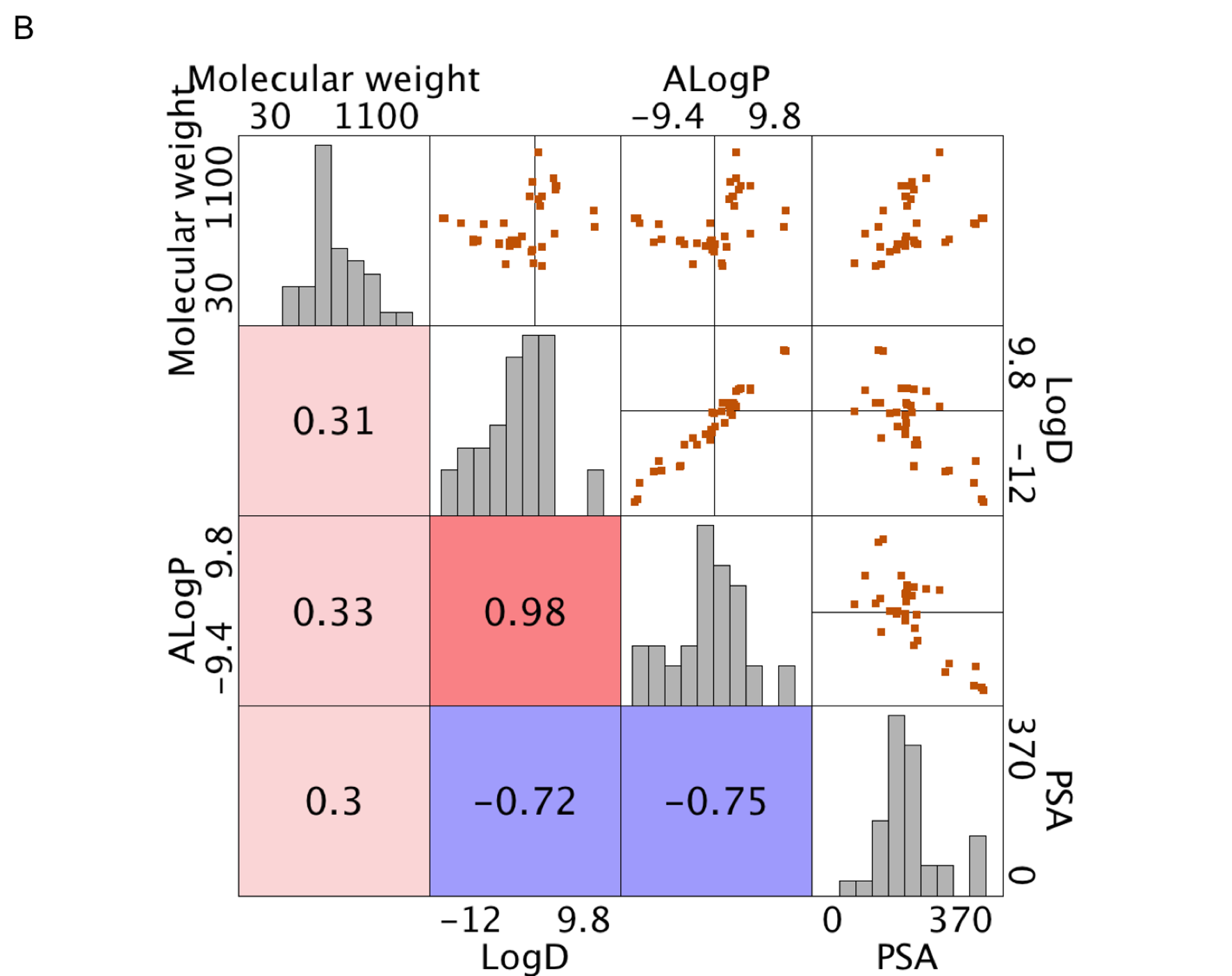

Supplement: Supplementary data — This document contains supplementary materials. [file mmc1.docx]
